# Successful Treatment of Innumerable Untreated Brain Metastases With Trastuzumab Deruxtecan in Chemotherapy-Naïve HER2-Mutated Non-Small-Cell Lung Cancer

**DOI:** 10.1155/crom/9936011

**Published:** 2025-04-23

**Authors:** Sara Young, Sean C. Dougherty, Angela M. DeRidder, Camilo E. Fadul, Ryan D. Gentzler

**Affiliations:** ^1^Department of Internal Medicine, University of Virginia, Charlottesville, Virginia, USA; ^2^Division of Hematology/Oncology, Department of Medicine, University of Virginia Cancer Center, Charlottesville, Virginia, USA; ^3^Department of Hematology/Oncology, Riverside Peninsula Cancer Institute and Infusion Center, Williamsburg, Virginia, USA; ^4^Division of Neuro-Oncology, Department of Neurology, University of Virginia, Charlottesville, Virginia, USA

**Keywords:** case report, HER-2 mutation, NSCLC, oncology, trastuzumab

## Abstract

In non-small-cell lung cancer (NSCLC), activating human epidermal growth factor receptor 2 (HER2) mutations are found in a small subset of patients and are associated with a higher incidence of brain metastases (BMETSs), conferring poor survival outcomes. Trastuzumab deruxtecan (T-DXd) was recently approved as a second-line agent for use in patients with previously treated, unresectable, or metastatic HER2-mutated NSCLC. We present a case of HER2-mutated NSCLC with BMETS, treated with T-DXd to defer whole-brain radiotherapy (WBRT) because of the concern of long-term neurotoxicity. He initially also received bevacizumab to address the cerebral edema, which allowed stopping corticosteroids. After the first two doses, the patient had remarkable clinical and imaging (brain and systemic) responses without progression after more than 1 year of treatment. T-DXd may be an effective and durable therapy for patients with HER2-mutated NSCLC with brain metastases in situations where intracranial disease would otherwise warrant WBRT. Clinical trials are needed to understand the efficacy and durability of T-DXd in NSCLC with BMETS and the optimal sequence of available therapies.

## 1. Introduction

Human epidermal growth factor receptor 2 (HER2) is a transmembrane glycoprotein receptor commonly overexpressed in breast and gastric cancer [1]. Treatment with HER2-targeted therapies improved clinical outcomes in these diseases, including patients with brain metastases (BMETSs). In non-small-cell lung cancer (NSCLC), both HER2 overexpression and activating HER2 mutations can occur. The activating mutations are less common and identified in approximately 1–6% of patients. HER2-mutated NSCLC generally presents with a more aggressive phenotype and a higher incidence of BMETS, conferring poor survival outcomes [1].

In August 2022, the Food and Drug Administration (FDA) granted accelerated approval for trastuzumab deruxtecan (T-DXd) in patients with previously treated, unresectable, or metastatic HER2-mutated NSCLC based on the DESTINY-Lung02 trial [1, 2]. All patients had received prior platinum chemotherapy. In this study, T-DXd treatment resulted in an objective response rate of 49%, median progression-free survival (PFS) of 9.9 months, and median overall survival (OS) of 19.5 months [2]. T-DXd is also FDA-approved for solid tumors with 3+ HER2 staining by immunohistochemistry (IHC). Previous studies have demonstrated CNS activity with T-DXd for breast cancer and HER2-mutated NSCLC with BMETS, but the clinical benefits and durability are not well established.

Several guidelines recommend radiation therapy, either whole-brain radiation therapy (WBRT) or stereotactic radiosurgery (SRS), for BMETS [3]. For patients with multiple BMETS, WBRT would be considered the standard of care, but there is concern about neurotoxicity in long-term survivors. Here, we report a case of HER2-mutated NSCLC with BMETS treated with T-DXd prior to platinum chemotherapy who was able to avoid radiation therapy, exhibit symptomatic improvement, and achieve durable CNS control.

## 2. Case Description

A 67-year-old male with a 25-pack-year smoking history presented with left focal motor seizure and expressive aphasia. Brain magnetic resonance imaging (MRI) demonstrated innumerable supra- and infratentorial peripheral enhancing lesions with edema, suggesting intracranial metastases. A body positron emission tomography-CT revealed a hypermetabolic left lung mass and mediastinal and supraclavicular lymph nodes compatible with lung cancer.

**Figure 1:**
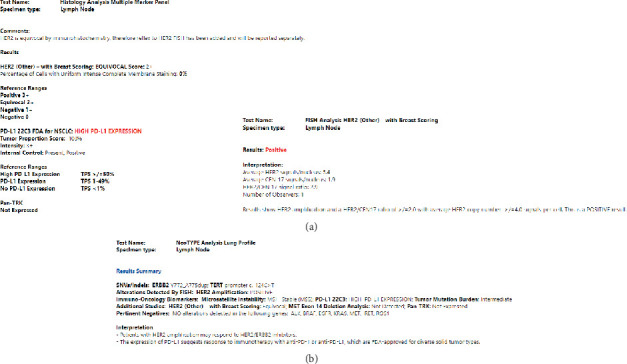
(a) IHC and FISH testing results. HER2 IHC with 2+ (equivocal) scoring. PD-L1 (22C3) with 100% tumor proportion score (high PD-L1 Expression), and FISH HER2 testing displaying HER2 amplification with an average HER2 copy number of 5.4 signals per cell (positive). (b) Next-generation sequencing results and overall pathology interpretation. HER2 sequencing testing displaying HER2 exon20 insertion, reported as “ERBB2 Y772_A775dup”. IHC: immunohistochemistry; FISH, fluorescent in situ hybridization; PD-L1, programmed death ligand 1.

An endobronchial biopsy of the lung mass revealed adenocarcinoma. PDL1 IHC (22C3) was 100%, and IHC testing for HER2 expression was 2+. Fluorescence in situ hybridization (FISH) testing demonstrated HER2 amplification with an average HER2 copy number of > 4.0 signals per cell (Figure 1a). Next-generation DNA-based sequencing testing later revealed a HER2 exon20 insertion, reported as “ERBB2 Y772_A775dup” (Figure 1b). This was not flagged as a variant of clinical significance.

**Figure 2:**
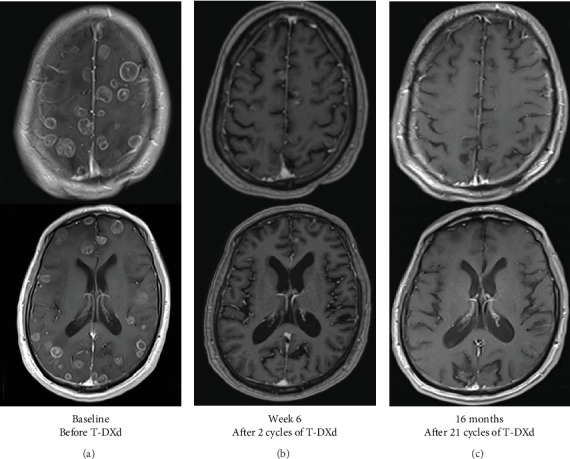
Axial view of MRI brain, postcontrast, showing innumerable metastases at (a) time of presentation after 1 cycle of pemrolizumab and prior to T-DXd, (b) decrease in size and number of lesions after two cycles of T-DXd, and (c) ongoing response after 16 months (21 cycles) of T-DXd. MRI, magnetic resonance imaging; T-DXd, trastuzumab deruxtecan.

He started pembrolizumab given high PDL1 expression. WBRT was recommended for the BMETS. He was referred to our center for a second opinion. A repeat brain MRI identified significantly increased size and number (> 60) of BMETS (Figure 2a). T-DXd was recommended, given the established efficacy in HER2-mutated NSCLC, to avoid potential long-term side effects associated with WBRT.

Pembrolizumab was discontinued after 1 cycle and T-DXd, 5.4 mg/kg, was started. Due to symptomatic corticosteroid-dependent cerebral edema, bevacizumab 7.5 mg/kg was also initiated. A repeat MRI brain obtained 6 weeks after T-DXd revealed remarkable improvement in cerebral edema and metastases (Figure 2b). Repeat CT chest revealed a decrease in size of the previously noted spiculated left lower lobe pulmonary nodule, from 3.5 to 2.1 cm, and complete resolution of previously enlarged mediastinal lymph nodes consistent with a partial response. The patient's neurologic symptoms resolved, allowing him to stop the corticosteroids and bevacizumab; he continued on single-agent T-DXd.

**Figure 3:**
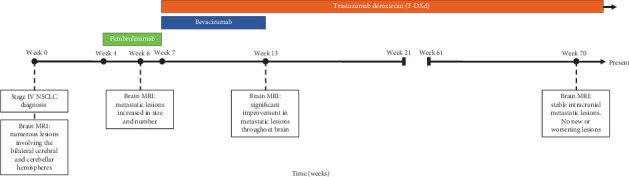
Timeline of patient's disease course to date, including times of pembrolizumab administration, 1 cycle (green box), bevacizumab administration, 4 cycles (blue box), and trastuzumab deruxtecan administration, 20+ cycles (orange box). Brain MRI's detailing response to therapy also noted.

After 16 months from baseline brain MRI (21 cycles of T-DXd), repeat brain MRI showed stable disease with no evidence of new lesions (Figure 2c). Repeat CT imaging also continued to show stable systemic disease; he continues to be treated with T-DXd (Figure 3).

## 3. Discussion

Radiation has been the mainstay for the treatment of BMETS. However, WBRT is associated with neurocognitive deterioration and reduced quality of life [4]. SRS is a more localized radiotherapy with fewer neurocognitive deficits and comparable survival after WBRT [4]. With a small number of BMETS, SRS is preferred over WBRT to minimize side effects [4]. However, SRS is not devoid of long-term adverse effects including cerebral necrosis [4]. In our case, we demonstrate systemic and CNS response of innumerable BMETS treated with T-DXd, avoiding the need for cerebral radiation.

Notably, our patient's cancer harbored a HER2 exon 20 insertion mutation that allowed for HER2-directed therapy. It is important to recognize the different testing methods available for HER2. Unlike breast or gastric cancer, HER2 mutations diagnosed by genomic sequencing are the biomarker of interest in NSCLC and do not always correlate with HER2 overexpression. HER2 sequencing is standard of care testing for NSCLC, recommended to be tested as part of a next-generation sequencing platform that includes other actionable genomic alterations.

T-DXd therapy was first evaluated for lung cancers with HER2 mutations. In a Phase I trial of T-DXd for patients with advanced solid tumors, 10 of 18 (55.6%) of patients with HER2-mutated NSCLC had an objective response and the median PFS was 11.3 months [5]. In the DESTINY-Lung01 trial, patients with metastatic HER2-mutated NSCLC demonstrated a median PFS of 8.2 months with a median OS of 17.8 months [6]. These studies were followed by the DESTINY-Lung02 trial exploring two different doses, which led to the FDA approval of the 5.4 mg/kg dose in lung cancers with HER2 mutations after prior platinum-based chemotherapy [2]. There are ongoing clinical trials with T-DXd as first-line therapy in HER2-mutated NSCLC, including the DESTINY-Lung04 trial (NCT05048797).

BMETSs are complex and challenging pathologies to treat with systemic therapy alone due to the presence of the blood–brain barrier, which limits drug delivery. For NSCLC with actionable oncogenic driver mutations, first-line targeted therapies with CNS activity can be effective for BMETS [1, 4]. Other mutations, such as HER2 and KRAS G12C, have agents with second-line approval status, but with lower response rates and less data on effectiveness for BMETS. Without targetable mutations, immune checkpoint inhibitors may have CNS efficacy in BMETS from NSCLC [4]. Of note, our patient did receive one dose of pembrolizumab prior to T-DXd initiation. As our patient possessed a remote smoking history (quit 25 years prior) and had only received one dose, we do not suspect the pembrolizumab contributed to the patient's longstanding or durable response. Ultimately, radiotherapy (WBRT or SRS) is the preferred treatment option when no CNS-active systemic therapies are available with the risk of neurotoxicity in long-term survivors [4]. Many trials of targeted therapy for lung cancer, including the DESTINY Lung02 trial, allowed for enrollment of patients with asymptomatic and untreated brain metastases. For patients with active, symptomatic brain metastases, the optimal treatment is unknown. Highly effective targeted therapies alone or in combination with local therapy such as SRS, surgical resection, or WBRT are available treatments [4].

In HER2-positive breast cancer with BMETS, the efficacy of T-DXd within the CNS is well established based on landmark trials. DESTINY-Breast03 demonstrated the superiority of T-Dxd when treating HER2-positive breast cancer with BMETS when compared to trastuzumab emtasine (percent of patients alive at 12 months without disease progression: 75.8% vs. 34.1%, respectively) [7]. There is emerging evidence of similarly beneficial CNS outcomes from T-DXd in NSCLC trials, specifically Destiny-Lung01 and Destiny-Lung02, where a pooled analysis demonstrated promising intracranial response of T-DXd in HER2-mutated NSCLC patients with BMETs [8]. This analysis showed a median intracranial response duration of 9.2 months as well as an intracranial objective response rate of 25% [8]. Importantly, our case expands upon this data to demonstrate over 12 months of a durable CNS response, suggesting long-lasting CNS penetration and efficacy of T-DXd in treating NSCLC BMETS are possible. Our patient received bevacizumab 7.5 mg/kg for the treatment of cerebral edema. It is possible that bevacizumb, which has clinical activity in lung adenocarcinomas, may have contributed to the initial response in the brain as well. However, this dose is lower than the usual 15 mg/kg used in NSCLC treatment and a limited duration of 4 cycles is less likely to explain the durable ongoing response. Additionally, this highlights the substantial systemic response of T-DXd, not limited to BMETS as seen in our patient's widespread disease reduction. In this chemotherapy-naïve patient, the enduring disease response to T-DXd suggests the need for further investigation into utilizing T-DXd as therapy for NSCLC with BMETS.

## 4. Conclusion

In a case with HER2-mutated NSCLC and innumerable symptomatic BMETS, we demonstrate early and durable clinical and imaging responses to treatment with T-DXd. In situations where CNS disease would otherwise warrant WBRT, T-DXd may be an effective therapy for BMETS from HER2-mutated NSCLC. Clinical trials are needed to determine the optimal treatment sequence and efficacy of T-DXd and other targeted therapies in BMETS from NSCLC.

## Data Availability

Data sharing is not applicable to this article as no new data were created or analyzed in this study.
